# Hydro-bio-geo-socio-chemical interactions and the sustainability of residential landscapes

**DOI:** 10.1093/pnasnexus/pgad316

**Published:** 2023-10-17

**Authors:** Peter M Groffman, Amanda K Suchy, Dexter H Locke, Robert J Johnston, David A Newburn, Arthur J Gold, Lawrence E Band, Jonathan Duncan, J Morgan Grove, Jenny Kao-Kniffin, Hallee Meltzer, Tom Ndebele, Jarlath O’Neil-Dunne, Colin Polsky, Grant L Thompson, Haoluan Wang, Ewa Zawojska

**Affiliations:** Advanced Science Research Center at the Graduate Center, City University of NewYork, New York, NY 10031, USA; Institute for Great Lakes Research and Biology Department, Central Michigan University, Mount Pleasant, MI 48858, USA; USDA Forest Service, Northern Research Station, Baltimore Field Station, Baltimore, MD 21228, USA; George Perkins Marsh Institute, Clark University, Worcester, MA 01610, USA; Department of Agricultural and Resource Economics, University of Maryland, College Park, MD 20742, USA; Department of Natural Resources Science, University of Rhode Island, Kingston, RI 02881, USA; Department of Environmental Science, and Engineering Systems and Environment, University of Virginia, Charlottesville, VA 22904, USA; Department of Ecosystem Science and Management, Pennsylvania State University, University Park, PA 16802, USA; USDA Forest Service, Northern Research Station, Baltimore Field Station, Baltimore, MD 21228, USA; School of Integrative Plant Science, Cornell University, Ithaca, NY 14850, USA; NOAA National Sea Grant Office, Silver Spring, MD 20910, USA; George Perkins Marsh Institute, Clark University, Worcester, MA 01610, USA; Spatial Analysis Laboratory, University of Vermont, Burlington, VT 05405, USA; Center for Environmental Studies, Florida Atlantic University, Davie, FL 33314, USA; Department of Horticulture, Iowa State University, Ames, IA 50011, USA; Department of Geography and Sustainable Development, University of Miami, Coral Gables, FL 33146, USA; Faculty of Economic Sciences, University of Warsaw, Warsaw, 00-241, Poland

## Abstract

Residential landscapes are essential to the sustainability of large areas of the United States. However, spatial and temporal variation across multiple domains complicates developing policies to balance these systems’ environmental, economic, and equity dimensions. We conducted multidisciplinary studies in the Baltimore, MD, USA, metropolitan area to identify locations (hotspots) or times (hot moments) with a disproportionate influence on nitrogen export, a widespread environmental concern. Results showed high variation in the inherent vulnerability/sensitivity of individual parcels to cause environmental damage and in the knowledge and practices of individual managers. To the extent that hotspots are the result of management choices by homeowners, there are straightforward approaches to improve outcomes, e.g. fertilizer restrictions and incentives to reduce fertilizer use. If, however, hotspots arise from the configuration and inherent characteristics of parcels and neighborhoods, efforts to improve outcomes may involve more intensive and complex interventions, such as conversion to alternative ecosystem types.

**Significance statement**

The paper presents major new insights into the multidisciplinary controls of nitrogen export (a widespread environmental concern) from residential landscapes. We conducted biogeochemical and social survey studies to identify locations (hotspots) or times (hot moments) with a disproportionate influence on this export. Results showed high variation in the vulnerability/sensitivity of individual parcels to cause environmental damage and in the knowledge and practices of individual managers. To the extent that hotspots are the result of management choices by homeowners, there are straightforward approaches to improve outcomes, e.g. fertilizer restrictions. If, however, hotspots arise from the configuration and inherent characteristics of parcels and neighborhoods, efforts to improve outcomes may involve more intensive and complex interventions, such as conversion to alternative ecosystem types.

## Introduction

Residential landscapes are an essential and increasing component of land use and land cover in the United States (1). The structure and function of these landscapes have important implications for environmental and human health and wellbeing (2, 3). There is particular interest in grass lawns which cover a significant portion of the area of residential landscapes and have diverse ecosystem services (e.g. esthetics, social cohesion, climate modification) and disservices (e.g. air and water quality) (4, 5).

Lawn ecosystems and residential landscapes are a clear, widespread, and important example of a social–ecological or coupled natural human system with implications for multiple components of sustainability: environment, equity, and economics (6). Analyses of these systems are complicated by spatial and temporal variation across various domains (e.g. hydro-bio-geo-chemical, homeowner management, economics, and public policy). While the immediate actions, private benefits, and costs of lawn management operate at the scale of the individual homeowner/lawn manager and residential parcel, environmental disservices occur offsite, downstream, and downwind (7). At the same time, the drivers of these actions are also multiscalar, as local decisions are influenced by climate, land use policies, neighborhood governance and norms, and intrahousehold dynamics (8). Moreover, there is tremendous variation in the inherent vulnerability/sensitivity of individual parcels to cause environmental damage and in the values, knowledge, and practices of individual managers (9–12). These complexities greatly hinder the production of policies that effectively balance environmental (water and air quality), economic (employment, retail sales, home values), and equity (value of green space, workplace hazards, quality of life) objectives (13, 14).

In addition to the conceptual complexities of evaluating lawn ecosystems and residential landscapes as coupled natural human systems, empirical studies have produced surprising and complex results. Multiple surveys of homeowners across the United States have shown that roughly half of homeowners apply fertilizer to their yards in any given year (15, 16), and that satisfaction with the quality of the environment in their neighborhood does not vary strongly with fertilizer use (17). In-depth interviews with homeowners across the United States suggest that people manage their yards for “beauty” as a priority, but with almost equal emphasis on “low maintenance” (18, 19). On the biophysical side of the coupled natural human system, studies have shown surprisingly high (similar to forests) retention of nitrogen added to lawn soils (20, 21). Studies have also shown that hydrologic and gaseous losses of nitrogen are not as high as expected, given that input rates can be high and are generally lower than for agricultural systems (22–24). Hydrologic dynamics in lawns and residential landscapes are also complex, with marked variation in soil compaction and infiltration capacity influencing the movement of water, nutrients, and pesticides off lawns and residential parcels (25–27). The microtopographical and edaphic variations in the landscape also influence atmospheric nitrogen removal and soil retention, primarily mediated by soil bacteria that perform denitrification, nitrification, and immobilization functions impacting the fate of nitrogen in watersheds (28). Adding these empirical complexities to the conceptual complexities of the flow of services and disservices discussed above further hinders the development of effective environmental policies for managing lawns and residential landscapes (29).

**Fig. 1.**
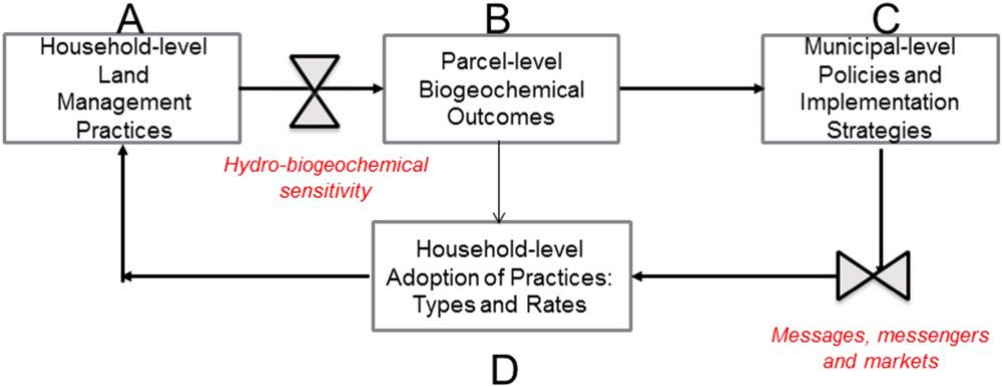
Interactions between hydrologic, biogeochemical, landscape structure, and social factors control nitrogen export from lawns and residential landscapes in a coupled natural human systems decision-making framework. Household-level management practices (A) influence nitrogen export (B), but these practices play out on a hydro-bio-geo-chemical template with varying susceptibility/vulnerability to nitrogen loss. Variation in export outcomes leads to the formulation of policies and implementation (C), but the adoption of these policies by homeowners/lawn managers (D) is influenced by social factors and communication approaches.

In this study, we investigated how interactions between hydrologic, biogeochemical, landscape structure, and socioeconomic factors influence nitrogen export from lawns and residential landscapes in a coupled natural human systems decision-making framework (Fig. 1). Our objective was to understand how this export is regulated by socioeconomic factors and behaviors on a hydro-bio-geo-chemical template with varying susceptibility/vulnerability to nitrogen loss. We were also interested in understanding how variation in export outcomes could lead to the formulation of policies and implementation, and how socioeconomic factors and communication approaches influence the adoption of these policies by homeowners/lawn managers. An overarching focus was to identify “hotspots” or “control points” of both nutrient fluxes and decision-making (30–33). Identifying these locations or topics of disproportionate influence can be key to developing transformative solutions to persistent challenges in sustainability science.

We addressed these objectives with coupled hydrologic, biogeochemical, and social science data for lawns and residential landscapes in the Baltimore, MD, USA, metropolitan area. Detailed measurements of water and nitrogen export were made on fertilized and unfertilized individual parcels in suburban, exurban, and institutional settings in the study region (34, 35). At the same time, extensive telephone and push-to-web surveys were carried out to quantify patterns of household fertilizer use and related behaviors such as lawn conversions to rain gardens or conservation landscaping (36, 37). Surveys also assessed households’ knowledge and attitudes toward fertilizer restrictions and the effect of policy changes on their willingness to adopt alternative lawn care and stormwater management practices (36). Results from these diverse data streams were evaluated and synthesized to identify locations or activities that could have a disproportionate influence on environmental disservices that could be the focus of policies directed at lawns and residential landscapes.

## Materials and methods

Hydro-bio-geo-chemical study lawns were located in the Piedmont region of Baltimore City and County, MD, USA (mean annual average temperature 13.0°C, 1,030 mm/y of annual precipitation) and encompassed exurban, suburban front yard, suburban backyard, and institutional lawns. The exurban lawns were in a neighborhood characterized by large homes and large (∼1 ha) lots, often dominated by lawns. The large lots included greater heterogeneity of topographic positions, including hollows and riparian zones that can act as nitrogen sinks. This exurban neighborhood is outside an “urban service boundary”, and therefore includes on-site (septic) sanitary effluent treatment based on septic tanks and leach fields that add nitrogen to the immediate landscape rather municipal sewage systems that transport waste to distal sewage treatment plants.

The suburban lawns were in a neighborhood served by municipal sewage systems dominated by small (∼0.1 ha) lots that typically have houses and other impervious surfaces occupying a substantial portion of the landscape. Rather than draining into local streams, most yards abut and drain into proximal impervious zones and curbs, short-circuiting the longer vegetated flowpaths in the exurban area. Institutional (land associated with hospitals, schools, libraries, auditoriums, and office complexes) lawns were located on a college campus.

Vegetation on the study lawns was dominated by cool-season perennial grasses: *Poa pratensis* (Kentucky bluegrass), *Festusca arundinacea* (tall fescue), and *Lolium perenne* (perennial ryegrass). To examine fertilizer effects on hydro-bio-geo-chemical properties of lawns, homeowners self-reported whether they fertilized in the last year (fertilized) or not (unfertilized). The institutional lawns were uniformly managed and fertilized (∼100 kg N/ha/y) by campus maintenance staff. We were focused exclusively on lawn fertilizers and did not address fertilizer applied to trees, gardens, or landscape plantings.

The potential of lawns to act as producers (sources) or consumers (sinks) of pollutant (reactive) nitrogen was assayed with measurements of their ability to absorb (infiltrate) water and their capacity for converting reactive nitrogen into nitrogen gas (34). We measured saturated infiltration rates using a Cornell sprinkling infiltrometer, and denitrification potential was measured with the short-term laboratory-based denitrification enzyme activity assay as described in detail in Ref. (34). Actual source/sink dynamics measurements were made during simulated rainfall events (35) in four separate field campaigns. Sampling encompassed comparisons of different topographic positions within both front and back yard lawns because prior research had identified different motivations, practices, and vegetation in front and back yards (9, 38–41).

Social science surveys accompanied the hydro-bio-geo-chemical measurements of nitrogen source and sink dynamics. As part of the US National Science Foundation-funded Baltimore Ecosystem Study Long Term Ecological Research program, spatially explicit household telephone surveys were conducted in 2003 (*n* = 1,508), 2006 (*n* = 3,312), 2011 (*n* = 1,636), and 2018 (*n* = 2,007) (17, 42–44). These surveys were distributed across socio-demographic segments representative of the dominant social groups at each time the survey was taken and included questions about environmental perceptions, environmental satisfaction, recreation practices, and demographics.

An additional push-to-web household survey (*N* = 3,836) was designed to collect data on lawn fertilizer application frequency as well as information on membership in homeowner and neighborhood associations, household demographics (e.g. education, income, age, children, pets outdoors, etc.), and housing and property characteristics (house size, house age, lot size) (45). Full methods and results are provided in Refs. (36, 37) and in the Online Supplementary Appendix. The survey was implemented over a random sample of single-family homeowners in Baltimore City and County, screened from a spatially explicit parcel-level tax assessor database to select owner-occupied residential lot sizes of 0.04 to 2 ha and ≥23 m^2^ of lawn area using high-resolution (1-m) land cover. The push-to-web sampling area included, as a subset, the neighborhoods evaluated by the hydro-bio-geo-chemical studies described above.

The push-to-web household survey supported multiple analyses of household behavior and policy support related to lawn care practices and fertilizer use. First, a two-step statistical model of fertilizer application behavior was estimated to account for household- and neighborhood-scale influences, parsed into informal and formal neighborhood effects (37). The model was estimated using *N* = 2,635 observations from the push-to-web survey that provided sufficiently complete response data on fertilizer use and household characteristics. Informal peer effects were quantified using novel measures that operationalize neighborhood conforming pressures based on housing and property characteristics of each parcel relative to those of surrounding neighbor parcels. The two-step model was estimated to understand and predict two sequenced lawn care decisions faced by households in the study area: whether to fertilize, and, if so, with what application frequency.

The push-to-web household survey also included supplemental questions on other types of actual and potential lawn care behaviors. These questions were divided over three distinct subsets, or versions of the survey questionnaire, supporting additional models of behavior and preferences (Online Supplementary Appendix). Within one of these three versions, discrete choice experiment (DCE) questions were included to evaluate (based on a latent class multinomial logit model) the probability that households would support alternative types of local programs or policies to reduce lawn fertilizer use, such as restrictions on fertilizer applications, tax surcharges on fertilizer products, educational programs for lawn assessments, and other mechanisms (46) (Online Supplementary Appendix).

Another version of the questionnaire included a set of DCE questions that elicited information on households’ willingness to convert lawns to alternative landscaping (e.g. rain gardens, conservation landscaping) in response to potential incentive programs for lawn conversion practices, with a focus on quantifying the multiple barriers (or “transaction costs”) that commonly inhibit program participation (36). Corresponding discrete choice models enabled predictions of whether households would enroll in potential cost-share programs that incentivize lawn conversion. The proposed programs varied over several program attributes, such as the percent cost-share paid and potential barriers in the enrollment process (e.g. finding a contractor, filing application paperwork, arranging a final inspection, paying full installation costs, and receiving a rebate later). A mixed logit, discrete choice model was estimated to predict enrollment (and hence lawn conversion) as a function of these program attributes, household demographics, and property characteristics (36).

Prior approval for human subjects research was obtained from the Institutional Review Board (IRB) of Clark University (FWA00000262), with the University of Maryland relying on the review and oversight authority of Clark University via an IRB Authorization Agreement. Approvals were obtained for all human subjects components of the study, including focus groups, pretesting and final survey implementation. Consent was documented for each respondent using an online consent form that preceded the survey.

## Results and discussion

Results of our multidisciplinary studies show that there is tremendous variation in both the inherent vulnerability/sensitivity of individual parcels to cause environmental damage and in the values, knowledge, and practices of individual homeowners/managers. These results have clear implications for sustainability policy. To the extent that hotspots of ecosystem disservices are the result of management choices by homeowners, there are multiple approaches to developing policies and programs to improve sustainability outcomes, e.g. fertilizer restrictions, education programs, soil testing, and expert lawn assessments. If, however, hotspots are not the result of management choices but rather arise from the configuration and inherent characteristics of parcels and neighborhoods, efforts to improve sustainable outcomes may involve targeting the conversion of a limited portion of lawn areas to other land covers, e.g. green infrastructure such as rain gardens and vegetated swales. Targeting such hotspots for conversion to alternative landscaping (e.g. rain gardens, ground covers, trees, or shrubs) (47) may be an efficient way to deal with disservices such as nitrogen flux. In some suburban situations, we suggest that changing 5–10% of lawn area to alternative landscaping might have a disproportionately large influence on neighborhood, landscape, or watershed flux.

### Hydro-bio-geo-chemistry matters

**Fig. 2.**
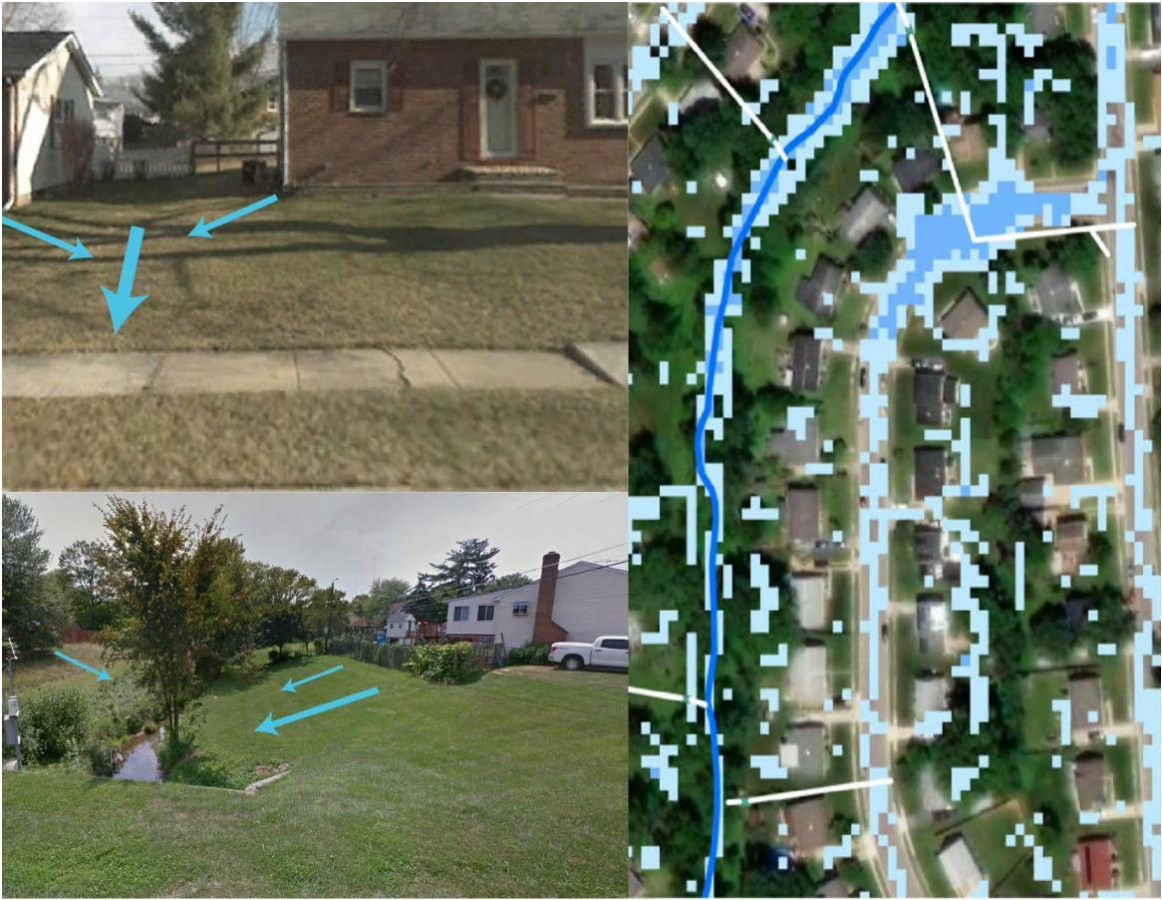
Human management interacts with hydrogeomorphic features both within and between parcel structures that greatly influence the offsite environmental impact of parcel management at large scales. These features include nonresidential parcels such as riparian areas, stream corridors, and drainage swales (in blue) and storm sewers (white) that cross parcel boundaries.

**Fig. 3.**
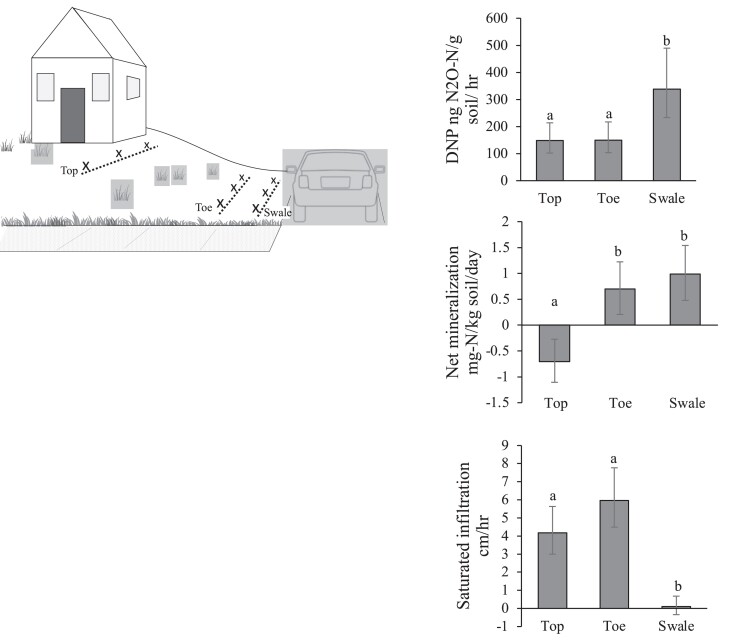
Significant functional variation in suburban front yards, driven by topography, influences sources (mineralization) and sinks (denitrification potential, DNP) of nitrogen and water (infiltration) within residential parcels. Different letters denote significant differences at *P* < 0.05. Error bars indicate ±1 S.E. Adapted from Suchy et al. (34).

A key finding from our work is that the configuration of natural and anthropogenic topographic and ecological features greatly influences the fluxes of water and nitrogen in lawns and residential landscapes. While the structure imposed by residential parcels is an obvious focal point for analysis and policy implementation (48), our analysis shows that there is significant variation in topography, hydrology, and biogeochemistry within parcels and residential landscapes (Fig. 2). Within a parcel, there is an inherent topographic structure driven by the desire to have water flow away from structures (Fig. 3). Just as in less human-dominated landscapes (e.g. forests, grasslands), topography affects sources and sinks of nitrogen and water within and export from the parcel. Low spots (toe slopes) within parcels receive water and nitrogen from upslope areas, and there is thus great interest in the ability of these spots to absorb water (infiltration) and to remove reactive nitrogen (denitrification or vegetation uptake). Our data show a challenging pattern in that the low spots have higher denitrification potential, but lower infiltration capacity. This pattern is particularly strong in suburban front yards that are directly adjacent to impervious surfaces (Fig. 3), suggesting that we may need to rethink the way residential parcels are graded or soils are managed to facilitate both denitrification and infiltration, either at the scale of the whole lot or through the strategic placement of a specialized feature such as a rain garden that is amended with permeable media (49).

**Fig. 4.**
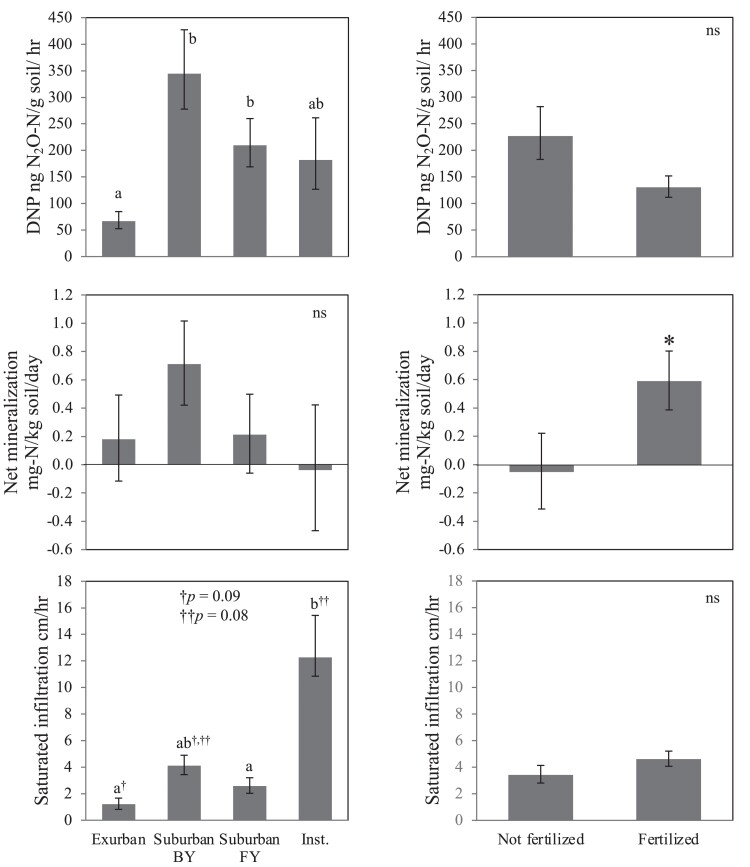
Significant functional variation in residential parcels, driven by neighborhood (exurban, suburban), front yard versus backyard (FY versus BY), institutional management (Inst.), and fertilizer application, influences sources (mineralization) and sinks (denitrification potential, DNP) of nitrogen and water (infiltration) within residential parcels. Different letters denote significant differences at *P* < 0.05. Error bars indicate ±1 S.E. Adapted from Suchy et al. (34).

Water and nitrogen dynamics also vary at larger scales. In our studies, residential parcels in suburban neighborhoods had significantly higher denitrification potential than exurban ones (Fig. 4). Parcel scale differences among neighborhoods could be caused by multiple factors, including topography, soil types, lot size (which influences the intensity of foot traffic and thus soil compaction and infiltration), and social norms (which influence management practices). The importance of minimizing foot traffic is supported by the high infiltration rates on institutional lawns, which likely get lower foot traffic than residential properties (Fig. 4).

#### The importance of fertilizer

Fertilizer application increases nitrogen supply and had no significant effect on infiltration rates or denitrification potential (Fig. 4). It is important to note that our exurban sampling sites were restricted to uplands (where the lawns are) and thus, our analysis does not account for high denitrification rates that likely occur downslope of residential lawns in riparian zones and other wet landscape features (50, 51). In our suburban neighborhood, lots are smaller and drain directly to impervious surfaces, reducing the potential for offsite removal.

**Fig. 5.**
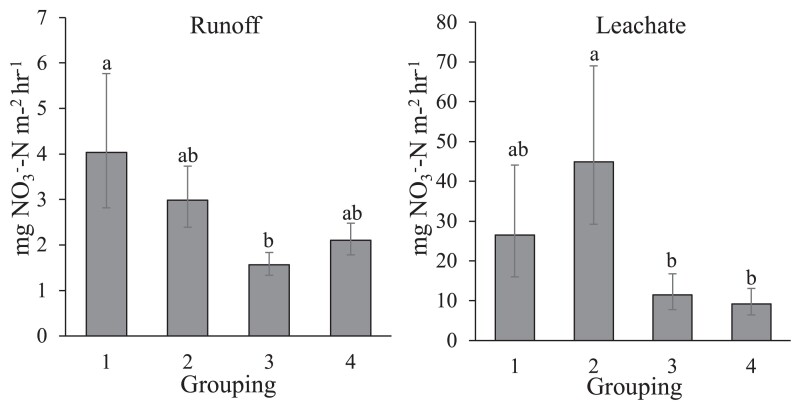
Nitrate flux in runoff and leachate for hydro-bio-geo-chemical “hotspot” groups of fertilized lawns. Different letters denote significant differences at *P* < 0.05. Group 1, which is comprised of sampling locations with low saturated infiltration rates (SInf) and low denitrification potential (DNP), has the highest potential to function as a hotspot of nitrogen export. Group 2 is high SInf and low DNP; group 3 is low SInf and high DNP; group 4 (lowest potential to function as a hotspot of export) is high SInf and high DNP. Error bars indicate ±1 S.E. Adapted from Suchy et al (35).

We grouped data from all sampling sites into four quadrants to indicate high or low potential to function as hotspots of nitrogen transport. Sites with high infiltration and high denitrification capacity had the lowest potential to function as nitrogen transport hotspots, while sites with low infiltration and low denitrification potential had the lowest potential to absorb or remove water and nitrogen and therefore had the highest potential to function as a nitrogen transport hotspot. This grouping helped to identify potential hotspots in the landscape; however, field measurements demonstrated that this potential was only realized on fertilized yards. Simulated rainfall events led to higher nitrate concentrations in both runoff and leachate in the two most vulnerable classes (i.e. low denitrification rates), but only in fertilized yards (Fig. 5). Thus, true hotspots and hot moment dynamics depend on an interaction between landscape characteristics and fertilizer application.

#### Fluxes and management implications of hydro-bio-geo-chemistry results

The final key finding from our hydro-bio-geo-chemistry analysis is that the amount of nitrogen flowing across yards and residential landscapes can be substantial in certain neighborhood settings. We collected and analyzed water flowing within and between parcels during a rainstorm in March 2019. A mass balance analysis combining nitrate concentrations measured in this water (0.75 mg N/L) with the estimated volume of runoff produced by a 5.0 cm rainfall event (likely to occur approximately once a year) suggests that such an event would export 0.22 kg N/ha from our suburban catchment (Dead Run 5) and 0.035 kg N/ha from our exurban catchment (Baisman Run). Based on prior empirical measurements of rainfall and flow, we assume that 58% of rainfall is converted to runoff in the suburban watershed, but during the dormant season the runoff to rainfall ratio could be even higher (52, 53).

The flux from the suburban catchment is noteworthy when compared to catchment-scale export flux estimates of 5.5 kg N/ha/y in Dead Run (54) and estimates of atmospheric deposition (6.6 kg N/ha/y) or fertilizer input (17.5 kg N/ha/y in Baisman Run, 19.2 kg N/ha/y in Dead Run). The analysis suggests that in a suburban catchment with sanitary sewers one significant, but not extreme, dormant season storm could export approximately 4% of the annual nitrogen flux, a considerable amount if water quality goals call for a 10–20% reduction in export. This contrasts with the minimal storm event contribution of nitrogen flux (0.5% of 6.4 kg N/ha/y of catchment flux) from hotspots and hot moments in yards in the Baisman Run exurban catchment, where septic systems dominate the nitrogen export flux through subsurface flowpaths. This runoff-centered analysis ignores the possibility of nitrogen in infiltrated water/leachate eventually being transported to the stream or being retained along the flow path.

The hydro-bio-geo-chemical analysis can be used to make a case for either fertilizer-based or hotspot-based approaches to reducing nitrogen exports from lawns and residential landscapes. Our analysis suggests that hotspots are “activated” by fertilizer input, so an alternative to a fertilizer ban could focus on spatial and temporal restrictions on use. Such a program would require identifying hotspots and variable management within and between parcels. Temporally based fertilizer restrictions are in place in several municipalities around the United States. However, the design and enforcement of these restrictions are complex, and their effectiveness has been mixed (55). Spatial restrictions, e.g. near-stream or lakeshores, have also been implemented in several locations, but again design and implementation of these restrictions are complex (56). Spatial approaches are appealing, however, as they may be an efficient way to deal with disservices such as nitrogen flux. For example, it might be possible to change the landscape structure and vegetation on the 5–10% of the lawns that have a disproportionately large influence on the neighborhood, landscape, or watershed flux.

### From hydro-bio-geo-chemistry to hydro-bio-geo-socio-chemistry

#### What do people know?

Our social science research directly addressed the knowledge, values, and actions (actual and potential) of homeowners in the Baltimore region. The first important finding is that accurate environmental knowledge on the impacts of lawn care practices is not widespread. Among the year 2018 survey respondents, 48% (926 of *N* = 2,007) said they do not live in a watershed, and an additional 6.17% (124) indicated that they do not know if they live in watershed, defined as “the drainage area to either a body of water itself or to its tributaries, such as the rivers and streams that eventually flow into it” (43). When push-to-web survey respondents were asked whether “waterways such as rivers, streams, ponds or lakes in your area are often negatively affected by nutrients such as nitrogen or phosphorus,” 61.2% (2,348 of *N* = 3,836) answered “I do not know” and 10.2% (392) answered “No” (Online Supplementary Table S3).

The lack of environmental knowledge included information specific to lawns. Push-to-web survey responses revealed that only 21.3% of homeowners (814 of *N* = 3,836 households) have had a soil test to determine if their lawns had excessive levels of nutrients (Online Supplementary Table S2). The Maryland Lawn Fertilizer Law has requirements for residential fertilizer application rates, but 69.0% of surveyed homeowners (2,646) were “not at all aware” of this statewide law implemented a decade ago (Online Supplementary Table S4). Another 7.9% of respondents (302) left this question blank. Only 2.7% (102) indicated that they were “highly aware” of the law.

The University of Maryland Extension Master Gardener Program and Bay-Wise Program provide extensive free educational resources for homeowners related to environmentally friendly lawn care and lawn conversion practices, yet 60.5% (2,322) and 62.7% (2,405) of homeowners indicated that they were “not at all aware” of these two programs (Online Supplementary Table S4). Moreover, fewer homeowners were either “moderately” or “highly aware” of these two programs (637 and 420 households for each program, respectively) than the number of homeowners that hired professional companies to apply fertilizer to their lawns (714; Online Supplementary Table S5). The juxtaposition of these two findings suggests that direct-to-consumer information on lawn care from private commercial firms may be a more common source of information on lawn care than the traditional Land-Grant University Cooperative Extension. This poses challenges and opportunities for fertilizer and nutrient reduction strategies. Private firms have incentives to encourage homeowners to buy services for lush green lawns, instead of considering the offsite and downstream impacts; however, greater consumer awareness may promote business opportunities for firms specializing in environmentally sustainable lawn care and landscaping.

This lack of knowledge creates a challenge for the flow of information from compartments B → C → D in our coupled natural human systems model (Fig. 1). While policy makers are aware of, and concerned about, the parcel-level biogeochemical outcomes that are affected by land management practices (as modified by hydro-bio-geo-chemical sensitivity), many homeowners are not. Even homeowners who are concerned about local water quality and other environmental conditions may not be aware of the potential impacts of their practices on these conditions. The dissemination and adoption of policies and implementation strategies by these homeowners may thus fundamentally depend on the messages, messengers, and markets used to engage and convince this audience for behavior change (57). If we increase homeowner awareness of the environmental impacts of lawn management practices and offer free soil tests, how might their behaviors change? How should outreach efforts be targeted at those who fertilize, especially in hydro-bio-geochemically sensitive areas? What happens when incentives do not work? What other types of policies or incentives would be most effective when seeking to change lawn care behaviors?

#### The importance of messengers, messages, and markets

Although our social science research clarifies the challenge related to a paucity of knowledge among typical homeowners, it also identifies potential ways to solve the hydro-bio-geo-socio-chemistry problem. First, we find that while homeowners are not particularly knowledgeable about environmental concerns in general, their actual practices are less intensive than expected. Several studies across the United States have found that approximately half of homeowners do not apply fertilizer in any given year (17). Similar results were found in the push-to-web survey in Baltimore City and County, with 47.9% of sampled homeowners reporting that they did not apply lawn fertilizer during the prior year (45). A similar proportion of nonapplying households (43.3%) can be inferred through analysis of other sets of survey questions (Online Supplementary Table S5). That said, the likelihood of fertilizer use and application frequency are significantly higher among identifiable household types. Newburn et al. (37) found that wealthier households with newer, larger homes are more likely to apply fertilizer and hire professional lawn care companies who apply fertilizer more frequently. These findings can help identify hotspots for lawn fertilizer application, thereby enabling policies and programs to be more effectively targeted.

The DCE on fertilizer policy choices also suggests that there would be public support for regulatory programs that would reduce lawn fertilizer use in the Baltimore area, in return for potential improvements in local river and stream conditions and reductions in lawn chemical exposure for children and pets. The latent class multinomial logit model reveals two distinct household types among the sample—class 1 (“lawn people”) households who care strongly about maintaining their lawns and class 2 (“pro-environment and pro-regulation”) households who favor policies to reduce lawn fertilizer use. Among other distinguishing factors, the likelihood of being in class 1 (relative to class 2) is higher for households who applied fertilizer more frequently during the past year, have larger shares of lawns in their parcels, and have lower assessed property values, ceteris paribus. On average, a household has a 54.47% probability of falling into class 1 and a 45.53% probability of falling into class 2 (Online Supplementary Table S8).

The probability of supporting policies that would reduce lawn fertilizer use varies between these two groups. Among both classes, support for restrictions was influenced by the extent to which the restrictions would lead to measurable improvements in the ecological condition of local streams due to reduced nitrogen runoff (*P* < 0.01) and reductions in lawn chemical exposure for local children and pets (*P* < 0.01). However, there were strong differences in the extent to which these two groups would support restrictions or surcharges on fertilizer use to obtain these desired outcomes. Class 1 households were less likely to support policies that include fertilizer surcharges (*P* < 0.01) and stringent restrictions on fertilizer application frequency (no more than 1 application per year; *P* < 0.01), and were more likely to support policies that offer free lawn assessments for households (*P* < 0.01). In contrast, households in class 2 displayed positive preferences for modest regulations (limited to no more than 3 applications per year; *P* < 0.01) and surcharges on lawn fertilizer (*P* < 0.05). Households in class 2 also had no statistically significant preference (positive or negative) for more stringent limits on fertilizer application frequency (limited to either 1 or 2 applications per year). Hence, results suggest heterogeneity in support for policies to reduce fertilizer use (Online Supplementary Table S8).

Despite this heterogeneity, results suggest that there would be majority support for many policies. To illustrate these predictions, Table S9 in the Online Supplementary Appendix shows predicted voting support among both household classes and the combined sample for a set of illustrative policies of the type that could be used to reduce residential lawn fertilizer application in Baltimore City and County. Model results predict majority support (>50%) for six of the seven illustrated cases, while two cases have predicted support from approximately 75% of the sample. In general, class 2 households have overwhelming support (over 90%) in all seven illustrated cases. Class 1 households have lower support, ranging from 8.6% (Option 7) to 55.4% (Option 3). Option 7, with the lowest predicted support over the combined sample (47.9%), restricts fertilizer use to no more than 1 application per year and includes a 20% surcharge for fertilizer purchases. Option 3 also restricts fertilizer use to no more than 1 application per year but simultaneously provides a program for free lawn assessments from certified lawn care experts (e.g. extension agents, master gardeners) who provide lawn care assistance to households. These results suggest robust support for a wide range of potential policies that would combine restrictions on fertilizer application frequency with surcharges on fertilizer products, but also show that support varies depending on the type of program that is proposed, particularly for households who fertilize more often (and are hence more likely to be in class 1).

The idea of targeting messages, messengers, and markets becomes even more important if we wish to combine fertilizer-based and hotspot-based approaches to reducing nitrogen exports from lawns and residential landscapes. Implementing hotspot-based approaches requires conversion of lawns to other residential landscaping, a considerably more complex change than fertilizer restrictions. Actual household adoption rates of lawn conversion practices, such as rain gardens, are typically low (58). Common programs to incentivize lawn conversions to rain gardens and conservation landscaping may not be optimally designed to encourage these conversions. Using the push-to-web survey data, mixed logit model results in Ref. (36) show that the enrollment barriers or “transaction costs” related to typical cost-share program requirements (e.g. finding a contractor, application paperwork for project design, final inspections, and delayed rebates) negate much of the incentive provided by typical cost-share payments and present a substantial barrier to widespread program participation. Predicted household enrollment in the presented cost-share programs more than doubles (from 23.2 to 49.7% of sampled households) when steps are taken to minimize these enrollment barriers (e.g. assisting homeowners with contracting and inspections), compared to the typical case in which these barriers are imposed. In such cases, local program resources typically allocated to provide higher cost-share payments in lawn conversion programs could be more effectively spent on strategies to attenuate these enrollment barriers. These results suggest that systematic changes to policy and incentive program design can have substantial implications for the probability that lawn conversions will occur in nitrogen hotspots (or anywhere else) (36).

## Conclusions

Our results support the idea that particular locations (hotspots) or times (hot moments) can have a disproportionate influence on nitrogen export in residential landscapes. We identified lawn areas and specific times within parcels, neighborhoods, and the metropolitan region as a whole with particular vulnerability to nitrogen export. This hydro-bio-geo-chemical finding is readily applicable to planning, assessment, and mitigation efforts to address nitrogen pollution problems in the Baltimore region, which drains to highly sensitive coastal waters in Chesapeake Bay.

The application of our findings is complex, however. The results could easily be used to support the idea of direct restrictions or bans on fertilizer use. Unfertilized yards were never significant sources of nitrogen export in our studies, and fertilizer addition created both hotspots and hot moments of export. Moreover, nearly half of homeowners do not routinely use fertilizer, while those who apply fertilizer are identifiable household types, e.g. those households with newer larger homes who are more likely to hire a lawn care company that applies fertilizer more frequently. A majority of households in our sample are willing to support restrictions on the frequency and amount of use. Still, support for restrictions is different than support for a total ban, which can motivate backlash, or resistance among those who strongly support the use of fertilizer. Indeed, negative reactions to fertilizer restrictions increase among nearly half of sampled households when the stringency of restrictions increases—suggesting that modest restrictions would be easier to enact (e.g. restricting to three applications per year). Support for restrictions is also substantially higher when simultaneously providing a program for free lawn assessments from certified experts. Perhaps an even more fundamental challenge to implementing fertilizer restrictions is the lack of awareness of current laws, policies, and programs related to fertilizer. Laws or public education programs will not have an effect if people are not aware of them.

The application of our findings on hotspots is even more complex. Conversion of particularly sensitive lawn areas to other types of “green infrastructure” could convert nitrogen source areas to nitrogen sinks, but support for such conversions is not strong, and there are significant barriers to the implementation of conversions, i.e. transaction costs. However, ideas about disproportionality suggest that overcoming these barriers could be an efficient way to improve the nitrogen performance of residential landscapes. If 5 or 10% of the watershed area could be converted from source to sink areas, it could have a large effect on this performance, but it depends on when and where those conversions take place within a watershed. These ideas raise questions about if traditional concepts of residential landscapes could be altered so that homeowners desire rain gardens or other landscape features designed to promote nitrogen removal (59) at toe slopes and other locations that serve as hotspots of nitrogen export.

An overarching conclusion from our work is that scientists and decision-makers require a better understanding of human perceptions and values, and how these affect behavior (60). Our initial ideas about fertilizer practices and attitudes were quite inaccurate, and our understanding of the barriers to lawn conversion was incomplete. Improving the sustainability of residential landscapes will require a deep understanding of the types of hydro-bio-geo-chemical processes studied here, but also of the hydro-bio-geo-socio-chemical interactions that ultimately regulate these processes. Listening to what people perceive and value and understanding how their perceptions and values influence their actions is key to improving the sustainability of residential landscapes.

## Supplementary Material

pgad316_Supplementary_Data

## Data Availability

The data in this manuscript are publicly available. Data not already available in Refs. (61–64) are included in the Online Supplementary material.
