# Supplementary material for: Hydro-bio-geo-socio-chemical interactions and the sustainability of residential landscapes
Source: PNAS Nexus. 2023 Oct 17;2(10):pgad316. doi: 10.1093/pnasnexus/pgad316 (PMC10581338; doi:10.1093/pnasnexus/pgad316)
Supplement: pgad316_Supplementary_Data [file pgad316_supplementary_data.zip › PNASNEXUS-PNASNEXUS-2023-00563R-s02.docx]

**
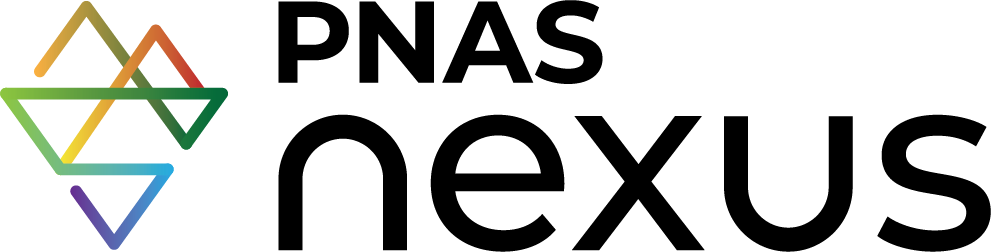
**

**Supplementary Online Data Appendix**

Hydro-bio-geo-socio-chemical interactions and the sustainability of residential landscapes

Peter M. Groffman, City University of New York, Advanced Science Research Center at the Graduate Center, New York, NY

Amanda K. Suchy, Institute for Great Lakes Research and Biology Department, Central Michigan University, Mount Pleasant, MI 48858 USA

Dexter H. Locke, USDA Forest Service, Northern Research Station, Baltimore Field Station, Suite 350, 5523 Research Park Drive, Baltimore, MD 21228, USA.

Robert J. Johnston, George Perkins Marsh Institute, Clark University, Worcester, MA 01610 USA

David A. Newburn, Department of Agricultural and Resource Economics, University of Maryland, College Park, MD 20742 USA

Arthur J. Gold, Department of Natural Resources Science, University of Rhode Island, Kingston, RI 02881, USA

Lawrence E. Band, Department of Environmental Science, and Engineering Systems and Environment, University of Virginia, Charlottesville, VA 22904 USA

Jonathan Duncan, Department of Ecosystem Science and Management, Pennsylvania State University, University Park, PA 16802 USA

J. Morgan Grove, USDA Forest Service, Northern Research Station, Baltimore Field Station, Suite 350, 5523 Research Park Drive, Baltimore, MD 21228, USA.

Jenny Kao-Kniffn, School of Integrative Plant Science, Cornell University, Ithaca, NY 14850

Hallee Meltzer, NOAA National Sea Grant Office, Silver Spring, MD 20910

Tom Ndebele, George Perkins Marsh Institute, Clark University, Worcester, MA 01610 USA

Jarlath O'Neil-Dunne, Spatial Analysis Laboratory, University of Vermont, Burlington, VT 05405

Colin Polsky, Center for Environmental Studies, Florida Atlantic University, Davie, FL 33314

Grant L. Thompson, Iowa State University, Department of Horticulture, 2206 Osborn Drive, Ames, IA 50011, USA

Haoluan Wang, Department of Geography and Sustainable Development, University of Miami, Coral Gables, FL 33146 USA

Ewa Zawojska, Faculty of Economic Sciences, University of Warsaw, Warsaw, Poland

Corresponding Author: Peter M. Groffman

Email: pgroffman@gc.cuny.edu

**README: Data and Code Appendix**

The model based on the discrete choice experiment data from Questionnaire Version B and presented in the *SI Appendix* was estimated using a custom code for discrete choice modeling developed in Matlab and available for download at https://github.com/czaj/DCE under CC BY 4.0 license. With this custom code installed, the model may be estimated using the simplified Matlab code listed below. This code enables estimation of the latent class model, the results of which are presented in Table S8 in the *SI Appendix*.

Within the code, data.mat refers to the data in Matlab format. The data is provided here in Excel format as fertilizer_dce_data.xlsx and can be directly imported to Matlab for the model estimation.

The following list shows the relationships between variable names in the data and the names used in the Supplementary Information (Table S7). In addition, the data includes variable “Vote”, which was used as the dependent variable in the model, indicating a respondent’s selection of an option in each discrete choice experiment task.

The data in the Excel file are presented in long form and are sorted first by questionnaire (six rows per questionnaire), then by binary choice question (two rows per question), then by choice alternative (one row for each choice alternative). This ordering can be used to create additional variables that identify questionnaires, questions and/or alternatives as needed to implement additional data analyses.

| **Variable name in the data** | **Variable name in the Supplementary Information** |
| --- | --- |
| Status_quo | Status quo |
| Applications_1 | 1 application allowed |
| Applications_2 | 2 applications allowed |
| Applications_3 | 3 applications allowed |
| Free_Assessment | Free lawn assessments |
| Surcharge | Fertilizer surcharge |
| River_Health | River & stream health |
| Chem_Exposure | Reduced chemical exposure |
| Cost | Cost |
| Fertilize_max | Number of fertilizer applications |
| Size_Acres | Parcel size |
| Percent_lawn_obj | Lawn share |
| Hire_Pro | Applying fertilizers via a company |
| DIY | Applying fertilizers by themselves |
| HOA | HOA |
| NHA | NHA |
| Pets | Outdoor pets |
| House_Age | House age |
| Market_value | House value |
| Male | Male |
| College4 | Bachelor’s degree |
| Master | Master’s degree |

**Code**

clear all

clc

global B_backup;

%% **************************** loading data ****************************

EstimOpt.DataFile = ('data.mat');

DATA = load(EstimOpt.DataFile);

%% **************************** data transformations ****************************

DATA.River_Health = DATA.River_Health/100;

DATA.Chem_Exposure = DATA.Chem_Exposure/100;

DATA.Surcharge = DATA.Surcharge/100;

DATA.Cost = DATA.Cost/100;

DATA.Market_value = DATA.Market_value/1000000;

%% **************************** model specification ****************************

DATA.Y = DATA.Vote;

DATA.Xa = [DATA.Status_quo, DATA.Applications_1, DATA.Applications_2, DATA.Applications_3, ...

DATA.Free_Assessment, DATA.River_Health, DATA.Chem_Exposure, DATA.Surcharge, -DATA.Cost];

EstimOpt.NamesA = {'Status quo';'1 application allowed';'2 applications allowed';'3 applications allowed';...

'Free lawn assessments';'River & stream health'; 'Reduced chemical exposure'; 'Fertilizer surcharge'; '-Cost (100 USD)'};

DATA.Xc = [DATA.Fertilize_max, DATA.Size_Acres, DATA.Percent_lawn_obj, ...

DATA.Hire_Pro, DATA.DIY, DATA.HOA, DATA.NHA, ...

DATA.Pets, DATA.Male, DATA.Master, DATA.College4, ...

DATA.House_Age, DATA.Market_value];

EstimOpt.NamesC = {'Fertilizer applications';'Parcel size (acres)'; 'Lawn share (%)';...

'Fertilize via a company';'Fertilize by themselves';'HOA'; 'NHA'; ...

'Outdoor pets'; 'Male'; 'Master’s degree'; 'Bachelor’s degree'; ...

'House age'; 'House value (million USD)'};

%% **************************** specifying input ****************************

DATA.filter = ones(size(DATA.Y)) == 1;

INPUT.Y = DATA.Y(DATA.filter);

INPUT.Xa = DATA.Xa(DATA.filter,:);

INPUT.Xc = DATA.Xc(DATA.filter,:);

%% **************************** sample characteristics ****************************

EstimOpt.NCT = 3; % Number of choice tasks per person

EstimOpt.NAlt = 2; % Number of alternatives

EstimOpt.NP = length(INPUT.Y)/EstimOpt.NCT/EstimOpt.NAlt; % Number of respondents

%% **************************** estimation ****************************

[INPUT, Results, EstimOpt, OptimOpt] = DataCleanDCE(INPUT,EstimOpt);

%% **************************** MNL ****************************

% Results.MNL.bhat = [-0.0489695287154918;-0.185400329817703;-0.119630533972979;0.140339401749478;0.193254469197610;4.11457024919275;1.01193347893008;-0.517530584009777;0.478464022770355];

Results.MNL = MNL(INPUT,Results,EstimOpt,OptimOpt);

%% **************************** LC ****************************

EstimOpt.NClass = 2; % number of latent classes

% B_backup = [1.59165492084479;-0.847760796432152;-0.272689872476004;0.207197444833177;1.07939430006453;7.71315402234628;2.81133794828122;-1.52479796123761;1.54369675452355;-1.87340054452057;0.0394612266364467;0.132526514803786;1.08334031357153;-0.166496980501904;11.6030388368941;2.03822738483769;2.17766447693651;1.04321982060289;0.818000199242293;0.0943536960173303;0.0181317828377871;1.01814013894294;0.0774987751284501;0.316882381346275;-0.146710858120877;-0.204784142498454;-0.194941555814171;0.439044386732880;-0.982898073394083;-0.800736580783001;-0.00590730602735560;-1.10942541629002];

Results.LC = LC(INPUT,Results,EstimOpt,OptimOpt);
