# Supplementary material for: Hydro-bio-geo-socio-chemical interactions and the sustainability of residential landscapes
Source: PNAS Nexus. 2023 Oct 17;2(10):pgad316. doi: 10.1093/pnasnexus/pgad316 (PMC10581338; doi:10.1093/pnasnexus/pgad316)
Supplement: pgad316_Supplementary_Data [file pgad316_supplementary_data.zip › PNASNEXUS-PNASNEXUS-2023-00563R-s01.docx]

**
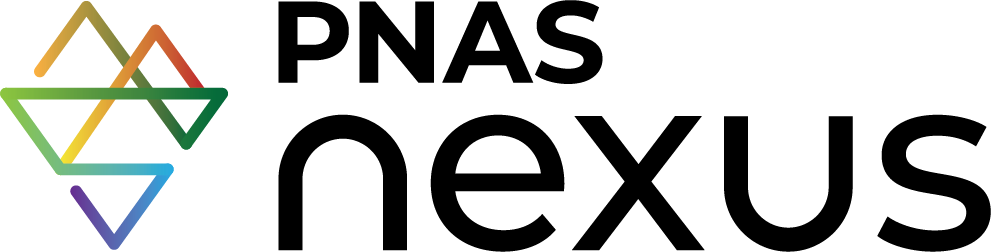
**

**Supplementary Information for**

Hydro-bio-geo-socio-chemical interactions and the sustainability of residential landscapes

Peter M. Groffman, City University of New York, Advanced Science Research Center at the Graduate Center, New York, NY

Amanda K. Suchy, Institute for Great Lakes Research and Biology Department, Central Michigan University, Mount Pleasant, MI 48858 USA

Dexter H. Locke, USDA Forest Service, Northern Research Station, Baltimore Field Station, Suite 350, 5523 Research Park Drive, Baltimore, MD 21228, USA.

Robert J. Johnston, George Perkins Marsh Institute, Clark University, Worcester, MA 01610 USA

David A. Newburn, Department of Agricultural and Resource Economics, University of Maryland, College Park, MD 20742 USA

Arthur J. Gold, Department of Natural Resources Science, University of Rhode Island, Kingston, RI 02881, USA

Lawrence E. Band, Department of Environmental Science, and Engineering Systems and Environment, University of Virginia, Charlottesville, VA 22904 USA

Jonathan Duncan, Department of Ecosystem Science and Management, Pennsylvania State University, University Park, PA 16802 USA

J. Morgan Grove, USDA Forest Service, Northern Research Station, Baltimore Field Station, Suite 350, 5523 Research Park Drive, Baltimore, MD 21228, USA.

Jenny Kao-Kniffn, School of Integrative Plant Science, Cornell University, Ithaca, NY 14850

Hallee Meltzer, NOAA National Sea Grant Office, Silver Spring, MD 20910

Tom Ndebele, George Perkins Marsh Institute, Clark University, Worcester, MA 01610 USA

Jarlath O'Neil-Dunne, Spatial Analysis Laboratory, University of Vermont, Burlington, VT 05405

Colin Polsky, Center for Environmental Studies, Florida Atlantic University, Davie, FL 33314

Grant L. Thompson, Iowa State University, Department of Horticulture, 2206 Osborn Drive, Ames, IA 50011, USA

Haoluan Wang, Department of Geography and Sustainable Development, University of Miami, Coral Gables, FL 33146 USA

Ewa Zawojska, Faculty of Economic Sciences, University of Warsaw, Warsaw, Poland

Corresponding Author: Peter M. Groffman

Email: pgroffman@gc.cuny.edu

**This PDF file includes:**

Supplementary text

Figure S1

Tables S1 to S9

SI References

**Supplementary Information Text**

**Push-to-Web Household Survey: Overview**

Primary results in the main text related to the knowledge, values, and actions (actual and potential) of homeowners were drawn from an address-based, push-to-web household survey implemented over a random sample of single-family homeowners in Baltimore City and County, Maryland, USA. This household survey was split into three distinct subsets, or questionnaire versions, each designed to elicit information on a different dimension of lawncare behaviors and preferences. Further details on sampling and response rates for each version are provided below.

The three questionnaire versions are denoted **Versions A**, **B** and **C**. All three versions collected data on lawn fertilizer application frequency along with information on membership in homeowner and neighborhood associations, household demographics (e.g., education, income, age, children, pets outdoors, etc.), and housing and property characteristics (e.g., house size, house age, lot size). Additional questions elicited information on associated lawn and landscape practices, including whether soil tests had been completed and lawn-related environmental knowledge. These survey-response data were combined with supplemental information on property characteristics for each responding household drawn from a spatially explicit parcel-level tax assessor database from the Maryland Tax and Assessment Office. The combined data were used to produce summary statistics on household fertilizer use and to estimate a two-step statistical model of fertilizer application behavior, as summarized in the main text and detailed in Newburn et al. [1].

Other questions differed across the three questionnaire versions and supported additional models on lawncare behavior and preferences reported in the main text. The first questionnaire version (**Version A**) included discrete choice experiment (DCE) questions designed to elicit information on households' willingness to convert lawns to alternative landscaping (e.g., rain gardens, conservation landscaping) in response to potential incentive programs for lawn conversion practices.^[[1]](#footnote-2)^ Methods and results from questionnaire Version A are detailed in Johnston et al. [2], leading to the corresponding conclusions in the main text.

A second questionnaire version (**Version B**) included an alternative set of DCE questions to evaluate households’ preferences over (and willingness to vote for) alternative types of local policies that could be used to reduce lawn fertilizer use and reduce corresponding nonpoint source pollution runoff. These policies included restrictions on fertilizer applications, tax surcharges on fertilizer products, educational programs for lawn assessments, and other mechanisms. Methods and results from this questionnaire version are not published elsewhere and are detailed below, leading to the summary findings and conclusions summarized in the main text.

A final and third version (**Version C**) did not include supplemental DCE questions of any type, and hence was only used to support analysis of households’ fertilizer application behavior and environmental knowledge as part of the analysis described above and in Newburn et al. [1].

Subsequent sections of this SI Appendix describe methods and results that support the conclusions reported in the main text, emphasizing material that has not been reported elsewhere. Methods and results for components of the household-survey analysis that are reported elsewhere [e.g., 1, 2] are not repeated here.

**Survey Design, Pretesting and Implementation**

As reported in both Newburn et al. [1] and Johnston et al. [2], the survey—including all questionnaire versions—was developed over a three-year period, in coordination with scientists from the Baltimore Ecosystem Study Long-Term Ecological Research site, with additional input from academics, government officials, and extension agents in the Baltimore region. Six focus groups were conducted to inform survey development and improve survey design. Additional survey pilot tests were conducted with 40 Maryland homeowners, with input from focus groups and pilot tests used to further refine survey design and content. These and other aspects of survey design and pretesting followed best practice guidelines [3, 4].

Prior approval for human subjects research was obtained from the Institutional Review Board (IRB) of Clark University (FWA00000262), with the University of Maryland relying on the review and oversight authority of Clark University via an IRB Authorization Agreement. Approvals were obtained for all human subjects components of the study, including focus groups, pretesting and final survey implementation.

The survey was implemented during November - December 2019 using an address-based, mixed-mode, push-to-web approach. Participation was solicited through mailed invitation letters that provided the website link (a condensed URL) to an online survey in Qualtrics. The addresses for mailed survey invitations were drawn randomly from the complete spatially explicit parcel-level tax assessor database from the Maryland Tax and Assessment Office. The sampling database was initially screened to select single-family, owner-occupied households with lot sizes from 0.1 to 5 acres, inclusive. To ensure that the property had a lawn, high-resolution (one meter) land cover data from the Chesapeake Conservancy was overlaid with the parcel boundary map. Parcels were screened to ensure that households receiving survey invitations had at least 23 square meters (250 square feet) of lawn area on their properties. This screening procedure yielded a total population of 153,978 households in the Baltimore study region, from which a random sample of 30,000 households was drawn for survey sampling.

Each of these 30,000 households received a personalized, mailed survey invitation letter, with a unique identification number and password. The initial invitation letter was followed, at weekly intervals, by a reminder postcard and final reminder letter. To increase response rates, those taking the survey were entered in a raffle for one of three $500 gift cards. This sampling procedure yielded a total of 3,836 responses (12.8% response rate), including both fully and partially completed questionnaires.^[[2]](#footnote-3)^ As introduced above, all questionnaire versions included questions on a core set of household lawncare practices and environmental knowledge, including questions on fertilizer application frequency. Of *N*=3,836 total survey returns, *N*=2,635 respondents provided sufficient response data for the two-step model of fertilizer application behavior, after accounting for item non-response on survey questions used as dependent and explanatory variables in the estimated models. Further details are found in Newburn et al. [1].

From the total mailing sample of 30,000 households, 13,000 received questionnaire Version A, yielding a usable sample size of *N*=1,748 completed questionnaires to support the analysis of lawn conversion behavior (13.5% response rate). Further details are in Johnston et al. [2]. An identical number of households (13,000) received questionnaire Version B, yielding *N*=1,657 completed questionnaires for analysis (12.8% response rate). Of Version B respondents, *N*=1,473 provided sufficient data on both DCE question responses and household characteristics to enable inclusion in the empirical analysis of fertilizer policy choices and preferences reported below. Finally, 4,000 of the 30,000 contacted households received questionnaire Version C that did not include supplemental DCE questions. These responses were included in the *N*=2,635 observations used to characterize households’ fertilizer application behavior and knowledge, but were not used for further analyses.

**Sample Characteristics and Representativeness**

Sample characteristics and representativeness for the household survey were evaluated to support the analysis in Newburn et al. [1]. To implement the analysis, observable parcel-level housing characteristics from the tax assessor database (e.g., house age, house size, lot size) and demographics (education, household income, age, unemployment rate, etc.) from the 2020 US Census are compared for respondents and non-respondents. Table S1 summarizes the means for these variables over respondents and non-respondents. As shown in Table S1, respondents and non-respondents were similar over most of these variables. Survey respondents had somewhat larger lot sizes and higher incomes, on average, than non-respondents, and were less likely to be minority. Given these modest differences between respondents and non-respondents, alternative preliminary models were estimated to evaluate whether the results reported in Newburn et al. [1] are robust to selection on observables. These preliminary models included an inverse probability weighted model that accommodated effects of sample selection on observables (i.e., non-response bias), following methods in Newburn and Alberini [5] and Rosenberg et al. [6]. Findings of these alternative models show that the conclusions reported in Newburn et al. [1] and the main text of this article are robust.

**Lawncare Practices, Knowledge and Lawn Fertilizer Application**

Newburn et al. [1] provide information on methods and results for the analysis of household lawn fertilizer use, supporting the findings reported in the main text. This prior article provides a summary of fertilizer use across sampled households, along with methods and results for the corresponding two-step model of fertilizer application frequency. To supplement these previously reported results, this section describes additional results described in the main text that are not found in Newburn et al. [1]. These results are drawn from all *N*=3,836 survey responses (combined questionnaire Versions A, B and C), with missing data noted as applicable.

Table S2 reports responses from the question, “Have you ever had a soil test done on your property?”, as discussed in the main text. As shown in the table, 21.3% of the sample (814 of 3,836) answered “yes,” either for lawn care or other purposes. Another 72.4% of the sample answered “no” (2,777 respondents), and the remaining 6.4% (245 respondents) either answered “I do not know” or did not answer the question.

Table S3 reports responses to a categorial question on whether “To the best of your knowledge, are waterways such as rivers, streams, ponds or lakes in your area often negatively affected by nutrients such as nitrogen or phosphorus?” As summarized in the main text, the majority of respondents (61.2%) answered “I do not know,” while 10.2% answered “No – most waterways are not affected by high or low nutrient levels.”

Table S4 reports responses to Likert-scale questions on familiarity with the Maryland Lawn Fertilizer Law, the University of Maryland Extension Master Gardener Program, and the University of Maryland Extension Bay-Wise Program. As summarized in the main text, the majority of respondents (over 60%) indicated that they were “not at all aware” the Maryland Lawn Fertilizer Law or the two University of Maryland Extension programs.

Table S5 reports responses to the question, “For each of these activities on your front lawn (in 2019), did you “do it yourself”, hire a professional, both or neither? “Do it yourself” includes activities done by you, family, or friends (any non-professional)” as discussed in the main text. As shown by these responses, 18.6% of responding households (714) indicated that they hire a professional company to apply lawn fertilizer. Comparing Tables S4 and S5, the total number of households who use a professional company to apply fertilizer (714) is greater than the number of households who are either moderately or highly aware of the two University of Maryland Extension programs (430 for the Bay-Wise Program; 637 for the Master Gardener Program). This suggests that professional lawncare companies in the private sector may be a more prevalent source of information for homeowners on lawn fertilizer application than experts in the public sector.

**Discrete Choice Experiment on Lawn Conversion**

Johnston et al. [2] provide information on methods and results for the DCE that evaluates households' willingness to convert lawns to alternative landscaping (e.g., rain gardens, conservation landscaping) in response to potential incentive programs for lawn conversion practices. Data for this analysis was drawn from questionnaire Version A. As discussed in the main text, this DCE enabled prediction of the probability that households would convert some or all of their lawn to alternative uses under alternative cost-share programs, with emphasis on the influence of enrollment barriers (or "transaction costs") that commonly inhibit program participation. Methods and results that support associated conclusions in the main text are described in Johnston et al. [2].

**Discrete Choice Experiment on Lawn Fertilizer Policy**

Questionnaire Version B included a DCE designed to evaluate households’ preferences and willingness to vote for alternative policies that could be used to reduce lawn fertilizer use and nonpoint source pollution runoff in the Baltimore metro area. One of the goals of this DCE was to estimate household preferences for different types of policy instruments that might be applied. As described above, DCEs estimate preferences using survey responses that identify choices that would be made under hypothetical but realistic choice scenarios. Each DCE question presents two or more choice alternatives (or options), with each alternative characterized by a set of attributes. By observing choices made by respondents over different types of policy options—characterized by different attribute levels—one can estimate the relative importance of each attribute to respondents’ policy preference and predict how households would vote if exposed to similar choices in actual, non-hypothetical settings. Here, DCE scenarios were designed around hypothetical but realistic multi-attribute programs that could be used to reduce residential lawn fertilizer use in the Baltimore metro area, leading to potential improvements in local river and stream conditions and reductions in lawn chemical exposure for children and pets. These methods and results have not been reported elsewhere and are described in the following supplementary text.

*Choice Experiment Design*

The DCE and its characterization of prospective policies were developed to reflect potential approaches to reduce residential lawn fertilizer applications applicable to urban, suburban, and exurban areas regions across the United States. These potential approaches included direct restrictions on fertilizer use, surcharges on fertilizer purchases, and free technical assistance from lawncare experts. The DCE questionnaire was designed to conform to best practices described in Johnston et al. [4], with input from experts, focus groups and pilot tests.

The DCE presented each respondent with a set of three binary choice situations, in which they were asked to evaluate (and vote for or against) a hypothetical policy that would affect residential lawncare in the Baltimore metro area. Each scenario presented a hypothetically binding, binary-choice question designed for incentive compatibility [7]. Respondents were instructed to consider each binary choice question as an independent, non-additive vote. Each program was described in terms of six attributes that characterized the regulatory or other approaches used to influence lawncare, environmental impacts, and the binding per household cost required to implement the policy. The status quo for each choice was described as Program A, which would lead to no change in policy, environmental condition or household cost. Program B was the presented policy alternative to the status quo, which would change these future conditions. All changes were described as taking place within 3 to 5 years.

Prior to presenting the choice questions, the questionnaire incorporated multiple elements to support valid preference elicitation, following recommended practices [4]. These included prompts and acknowledgement questions to emphasize payment and policy consequentiality, reminders of the household’s budget constraint, description of the decision rule linking responses and provision of the policy change (majority rule), an overview of valid reasons why a household might vote for either Program A or Program B, and a reminder that “Whatever your reasons, a vote for either Program A or Program B is legitimate.” To emphasize consequentiality, respondents were informed that a summary of anonymized survey results would be shared with government officials and non-profit organizations, to inform decisions on future policies in the Baltimore area. Other general dimensions of DCE and questionnaire design—which were shared across Versions A and B of the questionnaire—are described by Johnston et al. [2]. All survey components were subject to pretesting for clarity, salience and comprehension.

Table S6 describes the six attributes that characterized each choice alternative. Three of these described the approaches that would potentially be used to reduce residential fertilizer applications. The first attribute, *Fertilizer application restriction*, described whether new restrictions would limit the number of times per year that lawn fertilizer could be applied (yes or no), and if yes, the maximum number of applications that would be allowed per year (1, 2, or 3). Under the status quo, Maryland law does not limit the number of lawn fertilizer applications per year, so the number of applications by households is currently unrestricted. The second attribute, *Free lawn assessments*, indicated whether certified landscaping experts would be available to visit area homes free of charge, once per year (yes or no), to provide technical assistance. As described in the questionnaire, these experts would conduct lawn assessments and provide guidance to help households obtain desired lawn characteristics while reducing fertilizer and chemical use. The third attribute, *Fertilizer & chemical surcharge*, was described as an added percentage surcharge (from 0% to 30%) on prices that households would pay for lawn fertilizer and chemicals. The surcharge would also apply to those who hire companies to care for their lawns.

Two attributes characterized the environmental impacts of the proposed programs. The attribute *River & stream health* described the percentage (from 30% to 50%) of river and stream miles in the Baltimore metro area that are in fair, good or excellent health (on an excellent-good-fair-poor scale). This attribute and its status quo level was grounded in information and ratings provided in a report of biological impairments for the Baltimore Harbor Watershed [8]. The attribute *Reduce Chemical Exposure* described the decrease in exposure of local children and pets to lawn chemicals such as fertilizers, weed killers and pesticides (from 0% to 40%), compared to status quo levels. The levels for this attribute were informed by exposure estimates for children and pets in the literature [e.g., 9, 10].

The final attribute, *Household cost per year*, represented the hypothetically binding, annual cost per household (in unavoidable taxes and fees) that would be required to obtain the alternative policy scenario. Possible cost levels ranged from $0 to $325. These costs were described as being guaranteed to be spent only on the proposed program, in addition to any fertilizer and chemical surcharges that might be imposed. The cost of Program A (the no-policy status quo) was always $0.

The DCE was designed with visible choice sets [11], in which respondents were informed in advance of the possible range for each attribute that could appear in the choice questions. Prior to presentation of the three choice questions, the questionnaire provided background information on each attribute. An example DCE question is illustrated in Fig. S1.

The experimental design for the DCE (determining the combination of attribute levels that defined each choice question) was developed using a Bayesian D_b_-efficiency criterion for a choice model covariance matrix [12]. S-efficiency was also used to evaluate sample sizes required to estimate preference parameters for each assumed utility specification [12, 13]. Diffuse priors were applied [14], with signs based on information from focus groups, expert opinion, theory and findings from the literature. The resulting design included 48 profiles, or unique choice questions, blocked into 16 survey versions. Each questionnaire thus included 3 independent choice tasks.

The survey was implemented during November - December 2019 as described under *Survey Design, Pretesting and Implementation* above, yielding *N*=1,657 completed questionnaires. Of these, *N*=1,473 provided sufficient data to enable inclusion in the empirical analysis described below.

*Econometric Analysis*

Econometric analysis of the choice data was grounded in a standard random utility model and implemented using a latent class multinomial logit model in preference space [15]. Latent class models allow heterogeneity in preferences to be captured by probabilistic, discrete distributions. The model assumes that the utility of household $h$, within latent class $c$, from choosing policy alternative $p$ in choice task $j$ may be represented

$$U_{pjh}^{c}\left( \cdot\right)= {\boldsymbol{\beta}_{c}^{'}\boldsymbol{X}_{pjh}+\alpha_{c}F}_{pjh}+\varepsilon_{pjh} (1)$$

where $\boldsymbol{X}_{pjh}$ represents the vector of attributes that defines each policy (see above), $F_{phj}$ is the annual household cost of the alternative, and associated parameters to be estimated are given by $\boldsymbol{\beta}_{\boldsymbol{c}}^{\boldsymbol{'}}$ and $\alpha_{c}$. Within equation (1), utility is indexed by class $c$ ($c=\{1,2\ldots C\}$) to which a household belongs, such that estimated parameters are class-specific. This allows different preferences to be estimated for different household types (or latent classes). Variables in equation (1), drawn from the underlying DCE attributes described in Table S6, are defined in Table S7, along with corresponding means and standard deviations.

The probability that a household will belong to class $c$, and hence be characterized by the parameters estimated for that class, may be estimated as a function of observable household characteristics. Class membership probabilities are estimated as

$${}_{c}=\frac{\exp\left( {}_{c}+\boldsymbol{\varphi}_{\boldsymbol{c}}^{\boldsymbol{'}}\boldsymbol{V}_{h} \right)}{\sum_{m=1}^{C} \exp\left( {}_{m}+\boldsymbol{\varphi}_{\boldsymbol{m}}^{\boldsymbol{'}}\boldsymbol{V}_{h} \right)} (2)$$

where $\boldsymbol{V}_{h}$ is a vector of observable household characteristics, and parameters to be estimated are given by the conforming vectors and scalars $\boldsymbol{\varphi}_{\boldsymbol{m}}^{\boldsymbol{'}}$ and ${}_{m}$ for $m=\{1,2\ldots C\}$. Variables in vector $\boldsymbol{V}_{h}$ include observable demographic characteristics, parcel characteristics, prior use of lawn fertilizer, and neighborhood characteristics (Table S7).

The number of latent classes to be included in the model must be chosen by the researcher prior to estimation. This choice is typically made based on factors that include model performance and the consistency of the model specification and results with expectations derived from theory. Based on a consideration of preliminary results with alternative model specifications and theoretical expectations, the model characterized by (1) and (2) was estimated using two discrete classes. Given this specification, ${}_{c}$ and $\boldsymbol{\varphi}_{\boldsymbol{c}}$ are estimated for $c=1$. For identification, the parameters for class 2 are identically equal to zero (a reference category).

The model is estimated via maximum likelihood as a latent class multinomial logit model [15]. To promote model convergence, all continuous variables on the DCE attributes, including the variable on policy cost ($F_{phj}$), were divided (scaled) by 100 prior to estimation.^[[3]](#footnote-4)^ The corresponding parameter estimates are thereby interpreted as the relative marginal utility due to each 100-unit change in each continuous variable (e.g., each $100 change in cost or 100 percentage-point increase in river and stream condition). Dummy variables were not scaled. The econometric analysis was implemented in Matlab.^[[4]](#footnote-5)^

*Latent Class Model Results—Household Preferences for Fertilizer Policy*

Results of the latent class model are shown in Table S8. The data and code required to replicate these results are included as SI files for this article. The table includes two sets of primary results. The table first displays parameters of the utility function in (1), representing the preferences of each latent class. These estimates may be interpreted as the relative marginal utility (or strength of preference) given to each variable when choosing between policy Program A (the status quo) and Program B, accounting for variable scaling as described above. These preferences vary between the two latent classes, meaning that they vary between household types. Below these results, Table S8 displays estimated parameters of the class membership probability function, which are interpreted relative to class 2. These coefficients show the impact of each variable on the probability of a household being in latent class 1, as given by equation (2). The bottom of the table displays the average class probabilities and standard measures of model fit and performance. The model shows good overall performance and fit to the data, with model variables jointly significant at p < 0.001 and a McFadden pseudo-R^2^ of 0.26. Alternative preliminary models with different specifications reveal similar results.

Households in class 1 may be characterized colloquially and descriptively as “lawn people.” Estimated class probabilities suggest that a typical household in the sample has a 54.47% probability of falling into this class. Among other distinctions, the likelihood of being in class 1 is higher for households that previously applied fertilizer more frequently (and by themselves rather than via a lawncare company), have larger shares of lawns in their parcels, and have lower assessed property values and a smaller probability of having an advanced academic degree.

Compared to class 2, households in class 1 care relatively more about lawns and less about environmental outcomes. Households in class 1 hold positive and statistically significant preferences (marginal utilities) for improvements in river and stream condition and reductions in lawn chemical exposure for children and pets. These positive preferences notwithstanding, this class of households tends to hold negative preferences for policies that would affect lawncare. A positive and significant coefficient on the status-quo alternative specific constant (ASC) suggests that, all else equal, these households prefer to maintain the no-policy status quo. These households are less likely to support policies that include fertilizer surcharges and stringent restrictions on fertilizer application frequency (no more than 1 application per year), and are more likely to support policies that offer free lawn assessments for households. In sum, while households in this class support environmental improvements in general, they tend to resist policy actions that restrict or increase the expense of lawncare. However, even among this group, results show no statistically significant preference (positive or negative) for modest restrictions on fertilizer application frequency (limited to no more than 2 or 3 applications per year).

In contrast to class 1, households in class 2 may be characterized as “pro-environment and pro-regulation.” Estimated class probabilities suggest that a typical household in the sample has a 45.53% probability of falling into this class. As implied by the membership probability parameters for class 1, the probability of falling into class 2 is *lower* for households that previously applied fertilizer more frequently (and by themselves rather than via a lawncare company) and have larger shares of lawns in their parcels. These households tend to have higher assessed property values and higher education compared to those in class 1.

Like households in class 1, households in class 2 hold positive and statistically significant preferences (marginal utilities) for improvements in river and stream condition and reductions in lawn chemical exposure for children and pets. However, unlike those in class 1, households in class 2 tend to have *positive* preferences for modest regulations (limited to no more than 3 applications per year) and surcharges on lawn fertilizer and chemicals. Households in this class also have no statistically significant preference (positive or negative) for more stringent limits on fertilizer application frequency (limited to either 1 or 2 applications per year). These households tend to have systematic preferences to avoid the no-policy status quo, *ceteris paribus*, as revealed by a negative and statistically significant coefficient on the corresponding ASC. They also have no positive or negative preference for the provision of free lawn assessments.

*Predicting Support for Alternative Policy Options*

To illustrate the implications of these results for overall policy support, Table S9 shows the estimated probability that a household in each latent class ($c=\{1,2\}$) would vote in favor of a set of illustrative policy options, drawing from latent class model results in Table S8. The illustrated policy options draw from possible attribute levels shown in Table S6. A sum of these predicted support probabilities for each class, weighted by the predicted probability that a household falls into each latent class, allows the probability of a ‘yes’ vote to be estimated for the total sample. These results may be interpreted, equivalently, as the predicted proportion of the sample that would vote in favor of each policy. To better illustrate households’ willingness to support tradeoffs between lawncare restrictions and environmental improvements, *ceteris paribus*, all policies shown in Table S8 involve $0 change in annual taxes and fees.

As shown in Table S9 and summarized in the main text, the probability that a household will support policies to reduce lawn fertilizer use varies dramatically between the two classes. Class 2 households overwhelmingly support policies of this type, with greater than a 90% probability of a ‘yes’ vote for all illustrated policies. Similarly, high levels of support are found for all policies included in the underlying DCE experimental design. In contrast, the probability of a ‘yes’ vote is much lower for class 1 households, and the majority of illustrated policies fail to obtain majority support among this group. The starkest differences between these two classes are found for policy options such as Options 1 and 7, which restrict fertilizer use to no more than 1 application per year while simultaneously imposing surcharges on lawn fertilizer. In cases such as these, the probability of support among class 1 households is very low (13.9% and 8.6%), while the support probability for class 2 households is over 94%. However, a majority of class 1 households support Options 3 and 5 (55.4% and 54.8%), and the total support when combined for both classes is 74.7% and 75.1%. Option 3, for example, restricts fertilizer use to no more than 1 application per year but simultaneously provides a program for free lawn assessments from certified lawncare experts (e.g., extension agents, master gardeners) who provide guidance to the household to obtain the desired lawn appearance with reduced fertilizer and chemical use.

In most cases, the model predicts that most of the presented policy options would obtain majority support among the households in the sample. For six of the seven illustrated cases, the probability of a ‘yes’ vote is over 50% (corresponding to a prediction of majority support). For four of these cases (Options 2-5), the combined probability of support exceeds two-thirds of the sample population. These results suggest robust support for a wide range of potential policies that would combine restrictions on fertilizer application frequency with surcharges on fertilizer products. Similar conclusions emerge if one predicts parallel measures of voting support for other policy options included in the DCE experimental design.

**Fig. S1.** Example question from the fertilizer policy discrete choice experiment


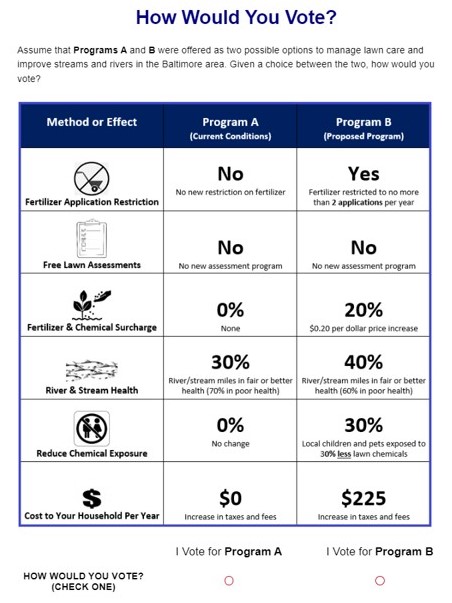


**Table S1.** Mean characteristics of survey respondents and non-respondents

| Variable | Respondents Mean | Non-respondents Mean |
| --- | --- | --- |
| Property characteristics |  |  |
| Lot size (acres) | 0.554 | 0.458 |
| House age (years) | 58.71 | 57.97 |
| House size (100 square meters) | 1.816 | 1.700 |
| Census tract-level characteristics |  |  |
| College degree proportion | 0.471 | 0.413 |
| Census block group-level characteristics |  |  |
| Racial proportions |  |  |
| White | 0.751 | 0.683 |
| Minority | 0.249 | 0.317 |
| Mean head of household age | 54.04 | 54.04 |
| Number of persons per household | 2.656 | 2.656 |
| Unemployment rate | 0.173 | 0.185 |
| Median household income ($1000) | 90.90 | 83.51 |
| Number of observations | 2,628 | 27,198 |

The total sample size for the analysis was *N* = 29,826. Of the 30,000 households in the survey mailing sample, 174 households (including 7 respondents and 167 non-respondents) were removed from the analysis in the table due to missing information on the census block group data.

**Table S2.** Summary Responses: “Have you ever had a soil test done on your property?” (*N*=3,836)

| **Answer** | **Frequency** | **Percent** |
| --- | --- | --- |
| Yes – for lawn care purposes | 624 | 16.3% |
| Yes – for other purposes | 190 | 5.0% |
| No – I have not | 2,777 | 72.4% |
| I don’t know | 147 | 3.8% |
| Missing | 98 | 2.6% |
| Total | 3,836 | 100% |

Frequency indicates the number of survey responses in each category.

**Table S3.** Summary Responses: “To the best of your knowledge, are waterways such as rivers, streams, ponds or lakes in your area often negatively affected by nutrients such as nitrogen or phosphorus?”

| Answer | Frequency | Percent |
| --- | --- | --- |
| Yes — many waterways are affected by high nutrient levels | 891 | 23.2% |
| Yes — many waterways are affected by low nutrient levels | 102 | 2.7% |
| No – most waterways are not affected by high or low nutrient levels | 392 | 10.2% |
| I don’t know | 2,348 | 61.2% |
| Missing | 103 | 2.7% |
| Total | 3,836 | 100% |

**Table S4.** Summary Responses: “How aware are you of the following?” (*N*=3,836)

| Awareness | Not at All Aware | Somewhat Aware | Moderately Aware | Highly Aware | Missing |
| --- | --- | --- | --- | --- | --- |
| The Maryland Lawn Fertilizer Law and its guidelines for home fertilizer use | 69.0%  (2,646) | 13.6%  (521) | 6.9%  (265) | 2.7%  (102) | 7.9%  (302) |
|  |  |  |  |  |  |
| The University of Maryland Extension Bay-Wise Program | 62.7%  (2,405) | 18.0%  (691) | 8.1%  (309) | 3.2%  (121) | 8.1%  (310) |
|  |  |  |  |  |  |
| The University of Maryland Extension Master Gardener Program | 60.5%  (2,322) | 14.7%  (565) | 9.4%  (362) | 7.2%  (275) | 8.1%  (312) |

Numbers in parentheses are the number of survey responses in each category.

**Table S5.** Summary Responses: “For each of these activities on your front lawn (in 2019), did you “do it yourself”, hire a professional, both or neither? “Do it Yourself” includes activities done by you, family, or friends (any non-professional).” (*N*=3,836)

| Activity | Do it Yourself | Hire a Pro | Both | Not Done | Missing | Total |
| --- | --- | --- | --- | --- | --- | --- |
| Fertilizing | 1,115 | 714 | 79 | 1,455 | 473 | 3,836 |
| Watering | 2,123 | 27 | 27 | 1,182 | 477 | 3,836 |
| Mowing | 2,528 | 931 | 150 | 4 | 223 | 3,836 |
| Insecticides | 573 | 566 | 84 | 1,949 | 664 | 3,836 |
| Weed control | 1,372 | 631 | 150 | 1,214 | 469 | 3,836 |

| Activity | Do it Yourself | Hire a Pro | Both | Not Done | Missing | Total |
| --- | --- | --- | --- | --- | --- | --- |
| Fertilizing | 29.1% | 18.6% | 2.1% | 37.9% | 12.3% | 100% |
| Watering | 55.3% | 0.7% | 0.7% | 30.8% | 12.4% | 100% |
| Mowing | 65.9% | 24.3% | 3.9% | 0.1% | 5.8% | 100% |
| Insecticides | 14.9% | 14.8% | 2.2% | 50.8% | 17.3% | 100% |
| Weed control | 35.8% | 16.5% | 3.9% | 31.7% | 12.2% | 100% |

Summary percentages with missing responses omitted

| Activity | Do it Yourself | Hire a Pro | Both | Not Done |
| --- | --- | --- | --- | --- |
| Fertilizing | 33.2% | 21.2% | 2.3% | 43.3% |
| Watering | 63.1% | 0.8% | 0.8% | 35.1% |
| Mowing | 75.2% | 27.7% | 4.5% | 0.1% |
| Insecticides | 17.0% | 16.8% | 2.5% | 58.0% |
| Weed control | 40.8% | 18.8% | 4.5% | 36.1% |

The top section of the table presents the number of responses in each category. The bottom two sections of the table present corresponding percentages, as a proportion of all responses and as a proportion of non-missing responses.

**Table S6.** Attributes and levels in the lawn fertilizer policy discrete choice experiment

| Attribute | Description | Attribute levels |
| --- | --- | --- |
| Fertilizer application restriction | Whether new restrictions limit the number of allowed fertilizer applications (yes or no), and if yes, the number of maximum allowed applications per year | No^SQ^  Yes, 1 application allowed;  Yes, 2 applications allowed;  Yes, 3 applications allowed |
| Free lawn assessments | Whether certified landscaping experts are available to visit homes free of charge once per year to conduct “lawn assessments” and provide guidance to help obtain desired lawn appearance while reducing fertilizer and chemicals | No^SQ^; Yes |
| Fertilizer & chemical surcharge | Added percent surcharge on prices of lawn fertilizer and chemicals, where revenue from the surcharge would pay for other parts of the policy | 0%^SQ^; 10%; 20%; 30% |
| River & stream health | Percent of river and stream miles in the Baltimore area that are in fair, good or excellent health (on an excellent-good-fair-poor scale) | 30%^SQ^; 35%; 40%; 45% |
| Reduced chemical exposure | Decrease in exposure of local children and pets to lawn chemicals, such as fertilizers, weed killers and pesticides, compared to current levels | 0%^SQ^; 15%; 30%; 45% |
| Cost | Household annual cost in a form of an unavoidable increase in taxes and fees guaranteed to be spent on the policy | $0^SQ^; $25; $50; $75; $125; $175; $225; $325 |

The levels associated with the status quo (no change) option are marked with a superscript SQ.

**Table S7.** Variables in latent class multinomial logit model utility and membership functions

| Variable | Definition | Mean (Std. dev.) |
| --- | --- | --- |
| *Variables describing discrete choice experiment options (derived from attributes in Table S6)* | | |
| Status quo | Binary variable with a value of 1 for the status quo option, 0 for the proposed policy option | 0.50 (0.50) |
| 1 application allowed | Binary variable with a value of 1 for the restriction allowing for a maximum of 1 application per year, 0 otherwise | 0.12 (0.33) |
| 2 applications allowed | Binary variable with a value of 1 for the restriction allowing for a maximum of 2 applications per year, 0 otherwise | 0.13 (0.33) |
| 3 applications allowed | Binary variable with a value of 1 for the restriction allowing for a maximum of 3 applications per year, 0 otherwise | 0.13 (0.33) |
| Free lawn assessments | Binary variable with a value of 1 for the availability of free lawn assessments, 0 otherwise | 0.25 (0.43) |
| Fertilizer surcharge^a^ | Continuous variable representing the percentage surcharge on prices of lawn fertilizer and chemicals | 7.53 (10.92) |
| River & stream health^a^ | Continuous variable representing the percentage of river and stream miles in fair, good or excellent health in the Baltimore area | 33.75 (5.44) |
| Reduced chemical exposure^a^ | Continuous variable representing the percentage decrease in exposure to lawn chemicals for children and pets | 11.24 (16.34) |
| Cost^a^ | Continuous variable representing per household cost per year (2019 USD) | 62.48 (96.33) |
| *Variables describing household and respondent characteristics* | | |
| Number of fertilizer applications | Continuous variable representing the number of fertilizer applications to lawns per year (from household survey responses)^b^ | 1.29 (1.84) |
| Parcel size | Continuous variable representing parcel size in acres (from Maryland tax assessor data) | 0.57 (0.78) |
| Lawn share | Continuous variable representing the percentage lawn share within a parcel (from high-resolution land cover data from the Chesapeake Conservancy) | 0.33 (0.19) |
| Applying fertilizers via a company | Binary variable taking a value of 1 for households hiring a lawncare company to apply fertilizers, 0 otherwise (from household survey responses) | 0.21 (0.41) |
| Applying fertilizers by themselves | Binary variable taking a value of 1 for households fertilizing by themselves, 0 otherwise (from household survey responses) | 0.32 (0.47) |
| HOA | Binary variable taking a value of 1 for households belonging to a homeowner association (HOA), 0 otherwise (from household survey responses) | 0.12 (0.33) |
| NHA | Binary variable taking a value of 1 for households belonging to a neighborhood house association (NHA), 0 otherwise (from household survey responses) | 0.28 (0.45) |
| Outdoor pets | Binary variable taking a value of 1 for households having outdoor pets, 0 otherwise (from household survey responses) | 0.50 (0.50) |
| House age | Age of the house in years as of 2019 (from Maryland tax assessor data) | 58.57 (27.70) |
| House value | Total assessed value of a house in million USD (from Maryland tax assessor data) | 0.31 (0.16) |
| Male | Binary variable taking a value of 1 for males, 0 otherwise (from household survey responses) | 0.68 (0.47) |
| Bachelor’s degree | Binary variable taking a value of 1 for respondents with a bachelor’s degree, 0 otherwise (from household survey responses) | 0.31 (0.46) |
| Master’s degree | Binary variable taking a value of 1 for respondents with a master’s or higher academic degree, 0 otherwise (from household survey responses) | 0.39 (0.49) |

Each of the 1,473 respondents faced three choice questions, which generates the total of 4,419 choice occasions. In 21 of them, selections of choice options were missing, and thus the statistics reported in the table are only for the 4,398 choice questions in which a choice of an option was made.

^a^ The variables on continuous attributes used in the discrete choice experiment were divided (scaled) by 100 prior to estimation. The statistics provided here show raw variable means and standard deviations prior to this scaling.

^b^ The survey elicited separate information on the number of fertilizer applications to the front and back yard. The number of fertilizer applications was calculated as the maximum of either front-yard applications or back-yard applications. For example, if a household made 2 applications to the front yard and no (0) applications to the back yard, this variable was assigned a value of 2.

**Table S8.** Latent class multinomial logit model results: fertilizer policy discrete choice experiment

|  | Class 1 | Class 2 |
| --- | --- | --- |
|  | Coefficient  (Std. err.) | Coefficient  (Std. err.) |
| *Utility function* |  |  |
| Status quo | 1.59*** (0.24) | -1.87*** (0.34) |
| 1 application allowed | -0.85*** (0.25) | 0.04 (0.25) |
| 2 applications allowed | -0.27 (0.22) | 0.13 (0.25) |
| 3 applications allowed | 0.21 (0.19) | 1.08*** (0.28) |
| Free lawn assessments | 1.08*** (0.17) | -0.17 (0.20) |
| Fertilizer surcharge^a^ | -1.52*** (0.58) | 2.18** (0.98) |
| River & stream health^a^ | 7.71*** (1.36) | 11.60*** (1.98) |
| Reduced chemical exposure^a^ | 2.81*** (0.72) | 2.04*** (0.71) |
| Cost^a^ | -1.54*** (0.15) | -1.04*** (0.14) |
| *Class membership probability function* |  |  |
| Constant | 0.82** (0.33) | 0.00 (fixed) |
| Number of fertilizer applications | 0.09* (0.05) | 0.00 (fixed) |
| Parcel size | 0.02 (0.09) | 0.00 (fixed) |
| Lawn share | 1.02*** (0.34) | 0.00 (fixed) |
| Applying fertilizers via a company | 0.08 (0.22) | 0.00 (fixed) |
| Applying fertilizers by themselves | 0.32** (0.15) | 0.00 (fixed) |
| HOA | -0.15 (0.21) | 0.00 (fixed) |
| NHA | -0.20 (0.14) | 0.00 (fixed) |
| Outdoor pets | -0.19 (0.12) | 0.00 (fixed) |
| House age | -0.01** (0.00) | 0.00 (fixed) |
| House value | -1.11** (0.52) | 0.00 (fixed) |
| Male | 0.44*** (0.13) | 0.00 (fixed) |
| Bachelor’s degree | -0.80*** (0.16) | 0.00 (fixed) |
| Master’s degree | -0.98*** (0.16) | 0.00 (fixed) |
| *Average class probabilities* | 54.47% | 45.53% |
| Log-likelihood at convergence | -2,249.59 |  |
| Log-likelihood at constants only | -3,047.73 |  |
| McFadden’s pseudo-R² | 0.262 |  |
| Ben-Akiva-Lerman’s pseudo-R² | 0.623 |  |
| AIC/*n*; BIC/*n* | 1.038; 1.084 |  |
| # of observations (*n*); # of respondents | 4,398; 1,473 |  |

^a^To promote model convergence, the variable was divided (scaled) by 100 prior to estimation. Parameters for these continuous variables are interpreted as marginal utilities per 100 units of change in the corresponding variable.

***, **, and * indicate 1%, 5%, and 10% significance levels, respectively.

**Table S9.** Predicted probability of policy support from the latent class multinomial logit model

|  | Illustrative policy options | | | | | | |
| --- | --- | --- | --- | --- | --- | --- | --- |
| Attribute | Option 1 | Option 2 | Option 3 | Option 4 | Option 5 | Option 6 | Option 7 |
| 1 application allowed | 1 | 0 | 1 | 0 | 0 | 0 | 1 |
| 2 applications allowed | 0 | 0 | 0 | 1 | 0 | 1 | 0 |
| 3 applications allowed | 0 | 0 | 0 | 0 | 1 | 0 | 0 |
| Free lawn assessments | 0 | 1 | 1 | 0 | 0 | 0 | 0 |
| Fertilizer surcharge | 10% | 30% | 0% | 0% | 0% | 30% | 20% |
| River & stream health^a^ | 40% | 40% | 45% | 45% | 45% | 30% | 35% |
| Reduced chemical exposure^a^ | 0% | 0% | 15% | 15% | 15% | 30% | 0% |
|  |  |  |  |  |  |  |  |
| Class 1 probability of ‘yes’ vote | 0.139 | 0.451 | 0.554 | 0.429 | 0.548 | 0.186 | 0.086 |
| Class 2 probability of ‘yes’ vote | 0.964 | 0.971 | 0.978 | 0.983 | 0.993 | 0.963 | 0.949 |
| Total probability of ‘yes’ vote (weighted class 1 & 2) | 0.515 | 0.688 | 0.747 | 0.681 | 0.751 | 0.540 | 0.479 |

Policy options are defined as combinations of attribute levels from the discrete choice experiment (see Table S6) and take levels according to the definitions in Table S7. Each presented policy option assumes a zero cost. The class-specific probabilities of a ‘yes’ vote are derived from the latent class multinomial logit model results reported in Table S8. The total probability of a ‘yes’ vote for the sample is calculated as the sum of probabilities for the two classes, weighted by the probability that a household falls into each class.

**SI References**

1. D.A. Newburn, R.J. Johnston, H. Wang, C. Polsky, T. Ndebele. Modeling multi-scale influences on household lawncare decisions: Formal and informal neighborhood conforming effects on fertilizer use. Forthcoming, *Landscape and Urban Planning*.
2. R. J. Johnston, T. Ndebele, D. Newburn, Modeling transaction costs in household adoption of landscape conservation practices. *Am J Agric Econ*, 105(1), 341-367 (2023).
3. D. Dillman, J. Smyth, L. Christian, L. Internet, Phone, Mail, and Mixed-mode Surveys: The Tailored Design Method. (John Wiley & Sons, 2014).
4. R. J. Johnston, K. J. Boyle, W. Adamowicz, J. Bennett, R. Brouwer, T.A. Cameron, W. M. Hanemann, N. Hanley, M. Ryan, R. Scarpa, R. Tourangeau, C. A. Vossler, Contemporary guidance for stated preference studies. *J Assoc Environ Resour Econ* 4(2), 319–405 (2017).
5. D. Newburn, A. Alberini, Household response to environmental incentives for rain garden adoption. *Water Resour. Res.*, 52(2), 1345-1357 (2016).
6. A. Rosenberg, D. Newburn, C. Towe, Household willingness to pay for stream restoration on public and private land: Evidence from the Baltimore metropolitan region. *J Am Water Resour Assoc.*, 59(2), 376-395, (2023).
7. R. T. Carson, T. Groves, Incentive and informational properties of preference questions. *Environ Resour Econ* 37(1),181–210 (2007).
8. Maryland Department of the Environment, Watershed report for biological impairment of the Baltimore Harbor Watershed in Baltimore City, Baltimore, and Anne Arundel counties, Maryland biological stressor identification analysis: Results and interpretation, Submitted to the Water Protection Division, U.S. Environmental Protection Agency, Region III, Baltimore, MD: Maryland Department of the Environment (2014).
9. M.G. Nishioka, R.G. Lewis, M.C. Brinkman, H.M. Burkholder, C.E. Hines, *et al.*, Distribution of 2, 4-D in air and on surfaces inside residences after lawn applications: comparing exposure estimates from various media for young children. *Environ. Health Perspect.,* 109(11), 1185-1191 (2001).
10. D.W. Knapp, W.A. Peer, A. Conteh, A.R. Diggs, B.R. Cooper, *et al.*, Detection of herbicides in the urine of pet dogs following home lawn chemical application. *Sci. Total Environ.,* 456, 34-41 (2013).
11. I.J. Bateman, M. Cole, P. Cooper, S. Georgiou, D. Hadley, and G.L. Poe. On visible choice sets and scope sensitivity. *J. Environ. Econ. Manag.* 47(1), 71-93, 2004.
12. R. Scarpa, J. Rose, Design efficiency for choice modelling. *Aust J Agric Resour Econ* 52(3), 253–282 (2008).
13. J.M. Rose, M.C.J. Bliemer, Constructing efficient stated choice experimental designs.” *Transp. Rev.* 29(5), 587-617 (2009).
14. S. Ferrini, R. Scarpa, “Designs with a priori Information for nonmarket valuation with choice experiments: A Monte Carlo study.” *J. Environ. Econ. Manag.* 53(3), 342-363 (2007).
15. W. H. Greene, D.A. Hensher, A latent class model for discrete choice analysis: Contrasts with mixed logit. *Transp. Res. B: Methodol.* **37(8)**, 681-698 (2003).

1. DCEs elicit information from survey respondents on choices or behaviors that would occur under hypothetical but realistic settings, where these settings often involve a set of prospective multi-attribute changes to environmental policies or programs relative to the status quo (Johnston et al. 2017). Data consisting of hypothetically binding choices over many scenarios, by many respondents, allows prediction of preferences and behaviors using econometric methods. [↑](#footnote-ref-2)
2. Information on the proportion of the 30,000 invitations that were deliverable is not available. Assuming that between 5-15% of these invitations were undeliverable (bad addresses)—a typical outcome for mailings of this type—the corresponding response rate for deliverable mailings would be between 13.5% and 15.0%. [↑](#footnote-ref-3)
3. This is a common approach for models of this type and has no substantive impact on the model results other than to promote convergence. [↑](#footnote-ref-4)
4. The model was estimated using a custom code for discrete choice modeling developed in Matlab and available for download at https://github.com/czaj/DCE under CC BY 4.0 license. With this custom code installed, the model may be estimated using the simplified Matlab code and data included as SI files. [↑](#footnote-ref-5)
